# A Step Toward Building User-Relevant Health Care Technology

**DOI:** 10.1016/j.mcpdig.2023.07.003

**Published:** 2023-08-08

**Authors:** Roopak Sinha

**Affiliations:** Department of Computer Science and Software Engineering, Auckland University of Technology, Auckland, New Zealand

Expensive ornaments or essential technology? A qualitative metasynthesis to identify lessons from user experiences of wearable devices and smart technology in health care,^1^ an article from an Australasian team of researchers, reports user experiences of wearable devices and smart technology in health care. It primarily focuses on how users and clinicians perceive the use of technology in health care. On the basis of 18 studies identified and analyzed after a qualitative metasynthesis, the authors identified 3 distinct themes of how technology may provide positive motivation and reassurance on the one hand but may also trigger resentment on the other.

This work is relevant as a step toward understanding why technology use has not yet become the default or mainstream option for managing people’s health. Technology promises efficiencies, scale, and cost-effectiveness. Technology can help balance inequities in access to health care through ubiquitous computers like mobile phones to capture insightful and timely information.^2^ This information can lead to more efficient and effective interventions. All these possibilities boost the returns on the stagnating investment in the health care sector. Although there is widespread acknowledgment of how technology can address critical challenges in health care, we are still struggling to integrate it into health care globally. User and clinician experience in technology use for health care is a primary factor in unlocking the role of technology in health care, and this factor is well studied in this article.

The qualitative metasynthesis presented in this article comprises a systematic literature search, quality appraisal drawing on the Joanna Briggs Institute qualitative systematic review methodology, and the 7-step meta-ethnography method. The research is well-designed, and the authors provide sufficient details, including artifacts that arise from each step in the process. These details strongly support the reproducibility of this work. Researchers may find the design of this research interesting and useful for training early postgraduate students on how to conduct systematic reviews.

This article’s novelty lies in its focus on qualitatively studying user experience and the thematic analysis of the sources, leading to identifying 3 distinct themes: technology as a motivator; reassurance from technology; and animosity toward technology. These are further divided into sub-themes. The results are rigorously analyzed, leading to a robust discussion of the findings, including identifying potential limitations, such as the relatively low number of studies covered by this review. There is a clear need for further research in the area of studying user experience. Other future areas of research originating from this work include studying how to improve data transparency, which is a clear deterrent to technology adoption in health care.

The broader scientific community will likely see parallels between health care and technology adoption initiatives in most other domains. For instance, human factors are extensively studied in software engineering^3^ and in developing complex cyber-physical systems such as automotive and large manufacturing systems.^4^ Studying such parallels can fuel inter-domain cross-pollination of ways to streamline technology adoption. Another exciting area that this article opens is understanding how individual experiences may combine to provide a social experience of technology. An interesting use case will be embedding support for good health and health care in future smart cities.

## Potential Competing Interests

The authors report no competing interests.
